# EEG correlates of sleep stages, sleep discrepancy, and insomnia: a comparative analysis of people with insomnia and healthy sleepers using spectral power and sample entropy

**DOI:** 10.3389/frsle.2026.1882890

**Published:** 2026-09-09

**Authors:** Joseph T. LeCheminant, Daniel B. Kay, Brent Feland, Chongming Yang, Jonathan Blotter

**Affiliations:** 1 Department of Mechanical Engineering, Brigham Young University, Provo, UT, United States; 2 Departments of Psychiatry and Neurology, University of Rochester Medical, Rochester, NY, United States; 3 Department of Exercise Science, Brigham Young University, Provo, UT, United States; 4 College of Family, Home, and Social Sciences, Research and Support Center, Brigham Young University, Provo, UT, United States

**Keywords:** band power, electroencephalography, insomnia, sample entropy, sleep discrepancy, sleep stages

## Abstract

This study investigated neurophysiological differences between individuals with and without insomnia across sleep stages and sleep discrepancy conditions using electroencephalography (EEG). EEG band powers (Delta, Beta) and entropy metrics (Sample Entropy and Multiscale Sample Entropy) were analyzed from each epoch of overnight polysomnography in 64 participants. Linear mixed-effects modeling was used to examine the influence of insomnia status, sleep stage, and electrode location on these EEG metrics. Results indicated that the EEG metrics were strongly correlated with physiological sleep stages but did not effectively distinguish self-reported sleep perception within the same polysomnographic sleep stage. Additionally, these metrics did not consistently differentiate individuals with insomnia from good sleeper controls across all conditions. Rather, individuals with insomnia exhibited regional patterns of Delta power during wakefulness, increased sample entropy during REM sleep, and decreased Beta power during positive discrepancy. These findings highlight physiological features associated with sleep stages, insomnia, and sleep discrepancy while emphasizing their complexity and the limitations of standard EEG metrics alone. Future work integrating physiological measures with self-report data may provide enhanced characterization of insomnia-related neurophysiological changes.

## 1 Introduction

Insomnia, characterized by difficulty initiating or reinitiating sleep, is a pervasive disorder that has been extensively studied using a range of neurophysiological measures. The hyperarousal model suggests that insomnia involves multiple forms of arousal, including somatic, cognitive, emotional, and central nervous system (CNS) arousal (Perlis et al., 2001a). Somatic arousal can be assessed by physiological measures such as heart rate, respiration, or electromyography. Cognitive and emotional arousal refers to excessive thoughts or feelings while trying to go to sleep and is typically evaluated through self-report. High frequency EEG has been arousal has been proposed as a measure of cognitive arousal. For example, Perlis et al. (2001a,b) reported increased high-frequency electroencephalogram (EEG) activity during sleep onset in individuals with insomnia. However, cognitive and affective activity are not exclusive to arousal and may be better conceptualized in the context of insomnia as heightened information processing into conscious awareness. Sleep processes also play a role in EEG patterns. This study sought to untangle EEG-derived measures of good sleepers and insomnia subjects to better understand the pathophysiology of insomnia.

EEG captures post-synaptic potentials of neuronal cells (Niedermeyer and Fernando, 2004) and provides insight into brain activity across sleep stages. Spectral power analysis is commonly used to quantify EEG activity. The spectral power of the Delta band (0–4 Hz) is associated with deep sleep, where post-synaptic potentials are more synchronized and have higher amplitude. In contrast, Beta band power (>13 Hz) is associated with wakefulness and active cognition, reflecting faster, desynchronized neural activity.

Sample entropy (SampEn) and its extension, multiscale sample entropy (MSE), offer additional quantitative analyses by measuring the irregularity and complexity of EEG signals, respectively (Costa et al., 2005). These measures provide further insight into the synchronization of post-synaptic potentials in the time domain. Research by Li et al. (2015) demonstrated that SampEn can effectively distinguish sleep stages, with lower entropy during deep sleep (synchronized potentials) and higher values during wakefulness and REM sleep (unsynchronized potentials, i.e., more signal irregularity) (Buzsákí and Draguhn, 2004). Similarly, Bruce et al. (2009) showed elevated values of SampEn in elderly individuals compared to middle-aged subjects during stages N2 and *R*, suggesting the potential to track sleep- and alertness-promoting mechanisms.

The distinction between regularity and complexity is crucial in understanding the information presented by SampEn and MSE (Goldberger et al., 2012). Sample entropy measures the irregularity of a signal by evaluating the presence of repetitive sequences. It reaches a maximum for completely random, uncorrelated signals (white noise) and a minimum for completely ordered signals (e.g., sine wave), though neither of these signals are complex (Costa et al., 2005). While SampEn effectively quantifies the irregularity of an EEG signal, it operates on the original time scale, limiting its ability to capture the full complexity of physiological processes.

Multiscale sample entropy extends this analysis by calculating SampEn at multiple time scales. The same SampEn calculation is performed on down-sampled versions of the original signal, revealing signal complexity as the persistence of irregularity across different time scales. For example, uncorrelated random noise yields high SampEn but low MSE, indicating irregularity without complexity. In contrast, correlated noise (colored noise) yields high SampEn and maintains high MSE across scales, reflecting both irregularity and complexity (Costa et al., 2005). These examples illustrate that SampEn alone may give misleading results (Costa et al., 2002). Jointly considering SampEn and MSE provides a more comprehensive understanding of the underlying dynamics of sleep stages and insomnia.

An insightful model of sleep-wake states proposed by Kay et al. describes insomnia and healthy sleep using three dimensions: sleep drive, arousal, and conscious awareness (Kay and Buysse, 2017). A normal balance of these indicators, such as high sleep drive with low wake drive, characterizes a healthy sleep or wake state. A conflicting combination, such as high conscious awareness and high sleep drive, represents an imbalanced state experienced by individuals with insomnia. Himes et al. (2020) opens discussion on quantifying sleep drive, arousal, and conscious awareness using Delta band power, Beta band power, and entropy, respectively. The EEG band power and entropy data in this study are considered in the context of this heuristic model of sleep-wake states, particularly those indicative of insomnia.

This study seeks to contribute by examining the EEG features in participants with insomnia compared to non-insomnia controls. Specifically, the study focuses on four key metrics: Delta and Beta band powers, SampEn, and MSE. The objectives are to explore neurophysiological differences between individuals with insomnia and good sleepers, and how these differences manifest across sleep stages and sleep discrepancy conditions. This research aims to contribute to a more comprehensive understanding of the EEG correlates of insomnia, with the potential to inform both diagnostic approaches and therapeutic interventions.

## 2 Methods

### 2.1 Participants

Data used for this analysis were obtained from two archival studies (Multimodal Neuroimaging STudy of Insomnia in relation to Research Domain Critiera [MNI_RDoC IRB#F16377] and Multimodal Neuroimaging of Insomnia during Non-rapid Eye Movement Sleep [MNI_NREM]) that included PSG polysomnography (PSG) recordings conducted by the BYU Department of Family Home and Social Sciences. Ongoing analysis of these data through the Sleep Psychology Archival Research Collaboration was approved by IRB2022-390.

The overnight ambulatory sleep studies were conducted in the participants' homes. Research assistants typically arrived 1.5 h before participants' habitual bedtime to begin electrode setup procedures, which took about 1–1.5 h. Participants were then allowed to sleep in their own beds throughout the night and returned the equipment the following morning. No environmental controls were imposed, and participants were instructed to follow their usual sleep routines. They were allowed to awaken spontaneously according to their normal schedules.

Data were collected between April 2017 and October 2019. Participants were recruited from BYU and the surrounding community through flyers and online advertisements. Clinical assessment confirming insomnia was performed using the Sleep Structured Clinical Interview for DSM-5 Sleep Disorders (SCID). Matching of cases and controls occurred only for the MNI_NREM protocol, not the MNI_RDoC. In the MNI_NREM protocol, matching was prospective but not strictly one-to-one; group sizes were balanced overall to avoid differences in final group composition.

The study included 64 participants with varying insomnia severity. A statistical power analysis indicated that this sample size was sufficient for the study. Insomnia severity was assessed using the Insomnia Severity Index (ISI), a self-reported questionnaire administered prior to participation. Insomnia status was classified as ISI ≥ 10 for individuals with insomnia and ISI < 10 for good-sleeper controls. Table 1 summarizes demographic information.

**Table 1:** Participant demographics summary.

Total *N*	64
Age	25 ± 6.7 years (range: 18–49)
Gender	Male: 32 (50%), female: 32 (50%)
ISI score	9.1 ± 6.3 (range: 0–22)
Insomnia status	Insomnia: 32 (50%), good sleepers: 32 (50%)

### 2.2 PSG data

EEG data were recorded at a sampling rate of 256 Hz from six channel locations defined by the international 10–20 system: F3, F4, C3, C4, O1, and O2, with reference channels M1 and M2.

Each subject's recording was processed and manually scored using ProFusion PSG™ 4 (Compumedics^®^) in accordance with the *AASM Manual for the Scoring of Sleep and Associated Events* (Berry et al., 2020). Prior to sleep stage scoring, a high-pass filter at 0.3 Hz was applied to remove linear trends, followed by a 35 Hz low-pass filter (Berry et al., 2020). Sleep stages were scored in 30-s epochs. Scoring of the PSG signal began just before lights out as participants settled into bed.

The PSG data include additional sleep parameters such as sleep onset latency (SOL), wake after sleep onset (WASO), and total sleep time (TST) according to AASM criteria. After filtering and scoring in ProFusion PSG™, EEG signals and sleep stage scores were exported for spectral and time-series analyses in MATLAB as described below.

### 2.3 Relative band power

Band powers were computed from EEG electrode in accordance with AASM-defined frequency bands (Berry et al., 2020):

- Delta: 0–3.99 Hz
- Theta: 4–7.99 Hz
- Alpha: 8–13 Hz
- Beta: >13 Hz

For each 30-s epoch, power spectral density (PSD) [μV^2^/Hz] was computed using Welch's periodogram with a Hamming window to minimize spectral leakage at epoch boundaries. Band power [μV^2^] was obtained by trapezoidal numerical integration of the PSD across each frequency band, as well as across the full bandwidth (0–35 Hz). Individual band powers were divided by the total band power to obtain unitless *relative* band powers. The correlation of relative band powers with sleep stage was assessed after excluding REM sleep. Without REM sleep, sleep stages and band powers exhibit a generally linear progression. Absolute band powers were also examined and yielded similar results; however, consistent with Zhao et al. (2021), relative band powers were found to be more sensitive for this analysis.

### 2.4 Sample entropy and multiscale sample entropy

SampEn and MSE were computed across each epoch and electrode. Each computation started with signal length *N* = 7, 680 samples (30-s epoch length at 256 Hz). Time series were coarse-grained using scale factors (τ) of τ = 1 through τ = 30. The shortest sample length was *N*/τ = 256 samples. Default parameter values of pattern length *m* = 2 and tolerance threshold *r* = 0.15 were used in the algorithm. The data were computed according to Costa et al. (2002), including a helper function written by Malik (2024).

Studies have utilized a variety of methods for reporting MSE from a SampEn vs. τ plot, as reviewed briefly by Himes et al. (2021). Since this study applies MSE to EEG data similar to those in (Himes et al., 2021), we adopt their approach of defining MSE as the average SampEn value from time scale τ = 20 to τ = 30. This was the range at which the MSE values flatten out for EEG data, and the average across that range produced a single MSE value to use for each epoch.

#### 2.4.1 Removing outliers

Since SampEn is sensitive to noise, outlier epochs were identified using a rolling interquartile range (IQR) method applied over 20-epoch windows. Epochs were rejected if SampEn was beyond a threshold of 1.5 times the IQR. Traditionally, artifact rejection is performed manually by visual inspection of the EEG, EOG, and EMG signals. However, due to the large dataset and automated approach was used to improve efficiency while maintaining consistency.

Morning diary entries for lights out and sleep latency were compared with PSG-derived sleep scores to determine discrepancy conditions. Participants reported “I actually tried to go to sleep at…” for lights out and “How long did it take you to fall asleep after you attempted to fall asleep?” to estimate SOL. Discrepancy condition labels were assigned to each sleep epoch for direct comparison with physiological sleep scores. Four conditions were defined: agreed wake (AW), positive discrepancy (PD), negative discrepancy (ND), and agreed sleep (AS) (see Table 2).

**Table 2:** Sleep discrepancy conditions.

Discrepancy condition	Agreed wake (AW)	Positive discrepancy (PD)	Negative discrepancy (ND)	Agreed sleep (AS)
PSG	Wake	Wake	Sleep	Sleep
Self-Report	Wake	Sleep	Wake	Sleep

A positive discrepancy corresponds to physiologically measured wakefulness during self-reported sleep. A negative discrepancy corresponds to physiologically measured sleep during self-reported wakefulness (i.e., overestimation of SOL).

Because self-reported awakenings after sleep onset were not timestamped, discrepancy analysis was limited to the sleep onset period. The discrepancy window began at the earlier of the two start times (self-report vs. PSG) and ended 20 min after the later onset time.

### 2.5 Statistical analysis

Correlation coefficients for each EEG metric and sleep stage were computed by converting sleep stages from a categorical to an ordinal numerical variable (W, N1, N2, N3 = 0, 1, 2, 3), representing a continuum from wakefulness to deep sleep. REM sleep (stage R) which does not follow this progression, was excluded from this analysis.

Linear mixed-effects (LME) models were used to investigate differences between insomnia and non-insomnia subjects across the EEG metrics at each electrode location and during each sleep stage. The models included insomnia status (binary: 0 = non-insomnia, 1 = insomnia), sleep stage (categorical: W, N1, N2, N3, R), and their interaction (Insomnia × Sleep Stage) as fixed effects. Subject ID was included as a random effect to account for repeated measures within subjects and inter-subject variability.

Rather than including electrode as a factor in a single model, separate models were fit for each electrode. This approach allows electrode-specific effects to be examined independently, acknowledging potential spatial differences in brain activity that may not be adequately captured by a global model.

Marginal means for the interaction between insomnia status and sleep stage were estimated with 95% confidence intervals (CIs). For each electrode, pairwise comparisons between insomnia and non-insomnia subjects were conducted within each sleep stage, and corresponding *p*-values were calculated. The models were estimated using the *lme4* and *emmeans* packages in R. Table 3 summarizes key characteristics of the mixed-effects models. This procedure was repeated for sleep discrepancy conditions, where the interaction term ‘Insomnia × Sleep Stage' was replaced with ‘Insomnia × Discrepancy Condition'.

**Table 3:** Mixed-effects model summary.

Model component	Description
Dependent variable	EEG metric (Delta, Theta, Alpha, Beta, MSE, and SampEn)
Fixed effects	Insomnia status (binary: 0 = non-insomnia, 1 = insomnia), sleep stage (categorical: W, N1, N2, N3, and R)
Random effect	Subject ID
Data Filter	Electrode location
Pairwise comparison	Insomnia vs. non-insomnia at each electrode and during each sleep stage
Confidence interval	95%
Significance level	*p* ≤ 0.05

## 3 Results

### 3.1 EEG metrics and sleep stages

**Figure 1:**
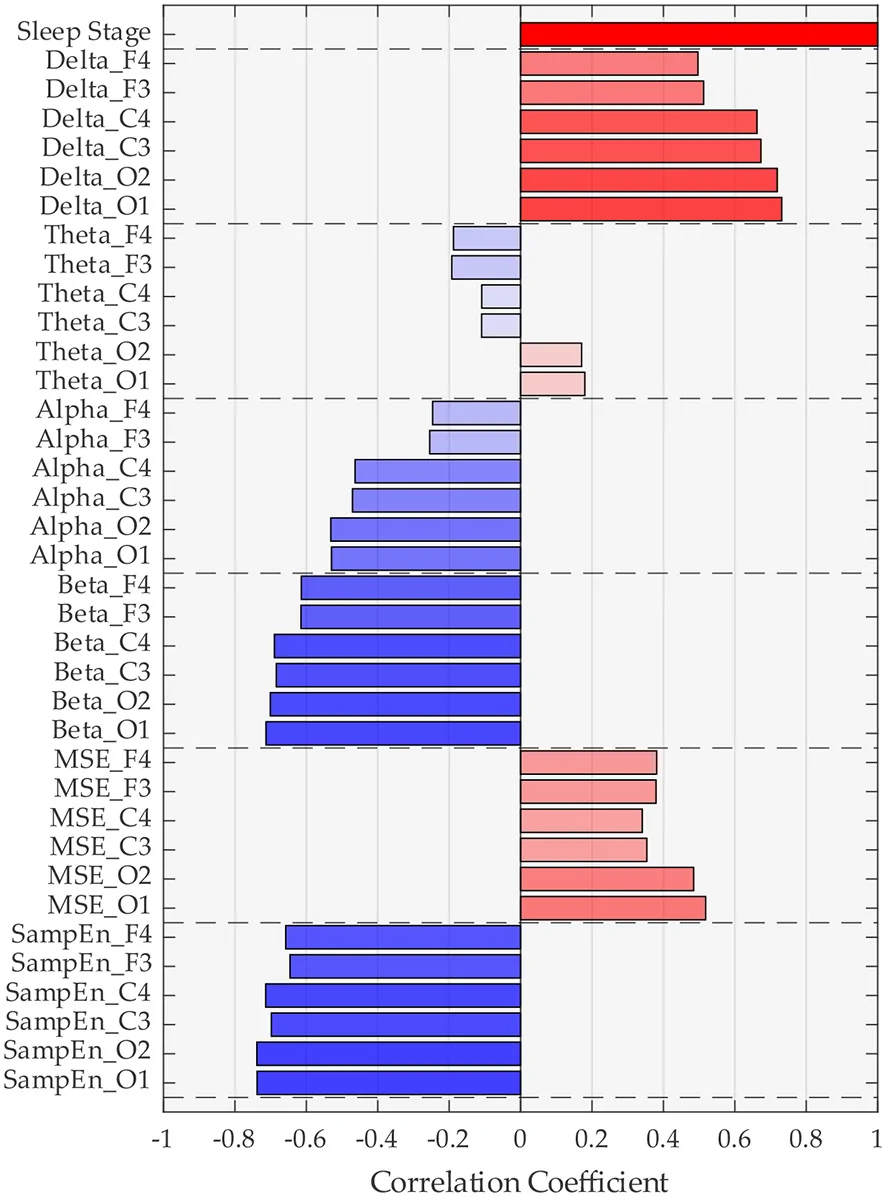
Correlation coefficients for EEG metrics (Delta, Theta, Alpha, Beta, SampEn, and MSE) from each channel (F3, F4, C3, C4, O1, and O2) with sleep stages. Excludes stage R. “Sleep Stage” correlation coefficient of 1.0 is included at the top of the figure for reference.

Figure 1 illustrates general trends of the EEG metrics used in this study in relation to depth of sleep, shown by correlation coefficients of each metric across channels. Positive correlation coefficients indicate an increase in the metric with increasing depth of sleep, while negative coefficients indicate a decrease. Delta and MSE exhibit positive correlations across all channels, indicating that low-frequency power and signal complexity generally increase from wakefulness to sleep. In contrast, Beta and SampEn show negative corrrelations across all channels, suggesting that high-frequency power and signal irregularity are greatest during wakefulness and decrease with increasing depth of sleep. Overall, occipital channels exhibit stronger correlations than other electrode locations.

Delta, Beta, and SampEn demonstrate the strongest associations with sleep stage. Theta shows mixed correlations across electrode locations, suggesting its presence across both wake and sleep states. Based on these results, Alpha and Theta band powers were excluded from further analysis due to weaker and less consistent correlations, while MSE was retained for joint analysis with SampEn.

**Figure 2:**
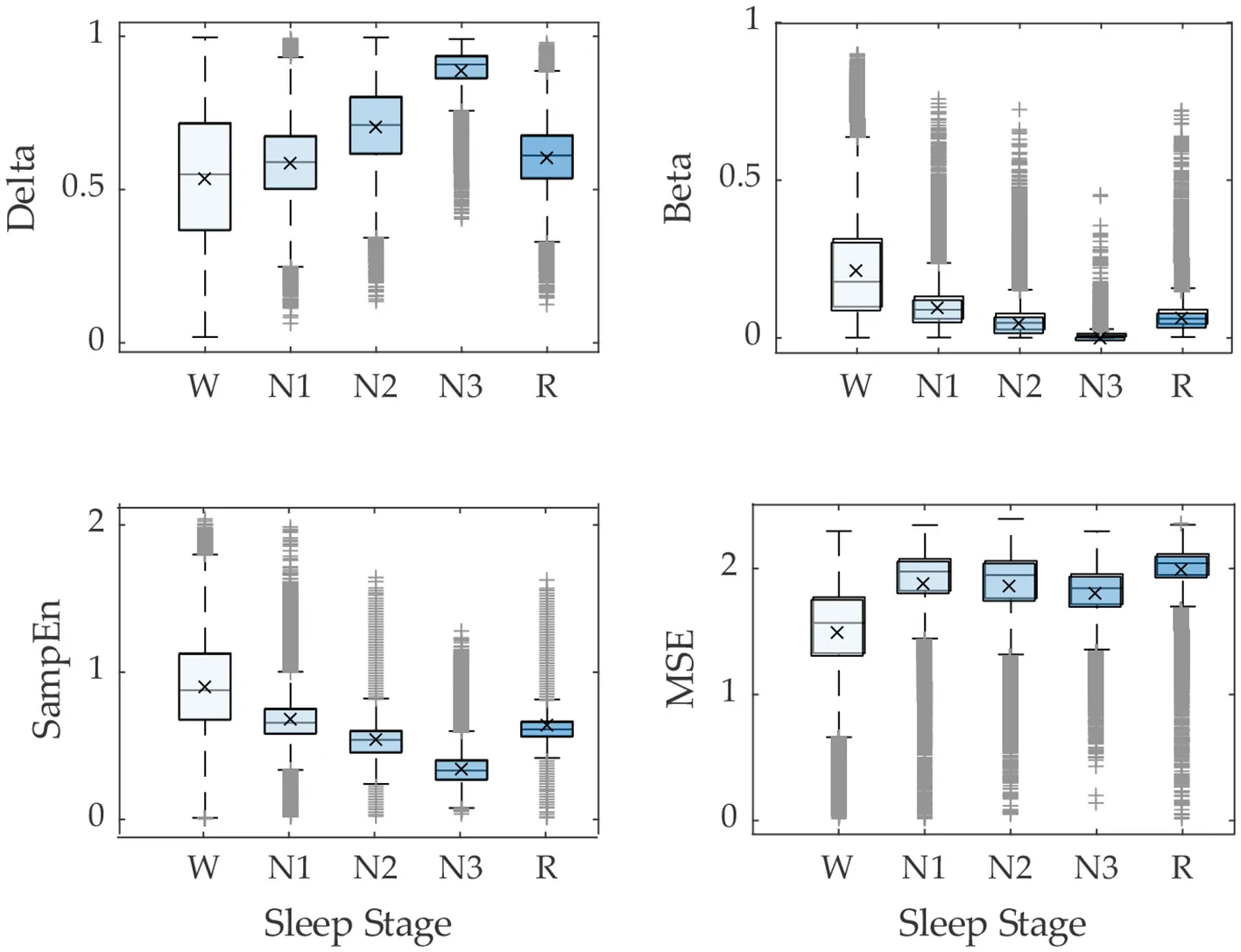
Relationship between EEG metrics (Delta, Beta, SampEn, and MSE) and sleep stages (W, N1, N2, N3, and REM). The plots include data from the entire subject population. Lower and upper box edges represent the 25th and 75th percentile, respectively, and whisker lengths are 1.5 times the interquartile range. The ‘x' markers are the sample means.

Figure 2 further demonstrates these relationships by plotting the EEG metrics from every epoch of each sleep stage in box and whisker charts. Each plot is inclusive of the data from all channels and includes stage R. Delta power means and medians consistently increase from stages W to N3, then fall during REM sleep. Beta and SampEn both consistently decrease until stage R.

The MSE data yield a trend unique to the other three, showing that the complexity of EEG signals is highest during light sleep and REM sleep. MSE initially increases from wakefulness to light sleep (stage W to N1), decreases from stages N1 to N3, then increases again in stage R.

Each box and whisker plot includes a large spread of outliers, highlighting the difficulty of EEG signal processing. Without focusing on the means and medians, the trends may be less clear. It is reasonable for EEG data from wakefulness epochs to be noisier and more unpredictable. The interquartile range and range of spread is consistently smallest during stage N3, which is to be expected during epochs with the highest fidelity signal.

### 3.2 EEG metrics and sleep discrepancy

**Figure 3:**
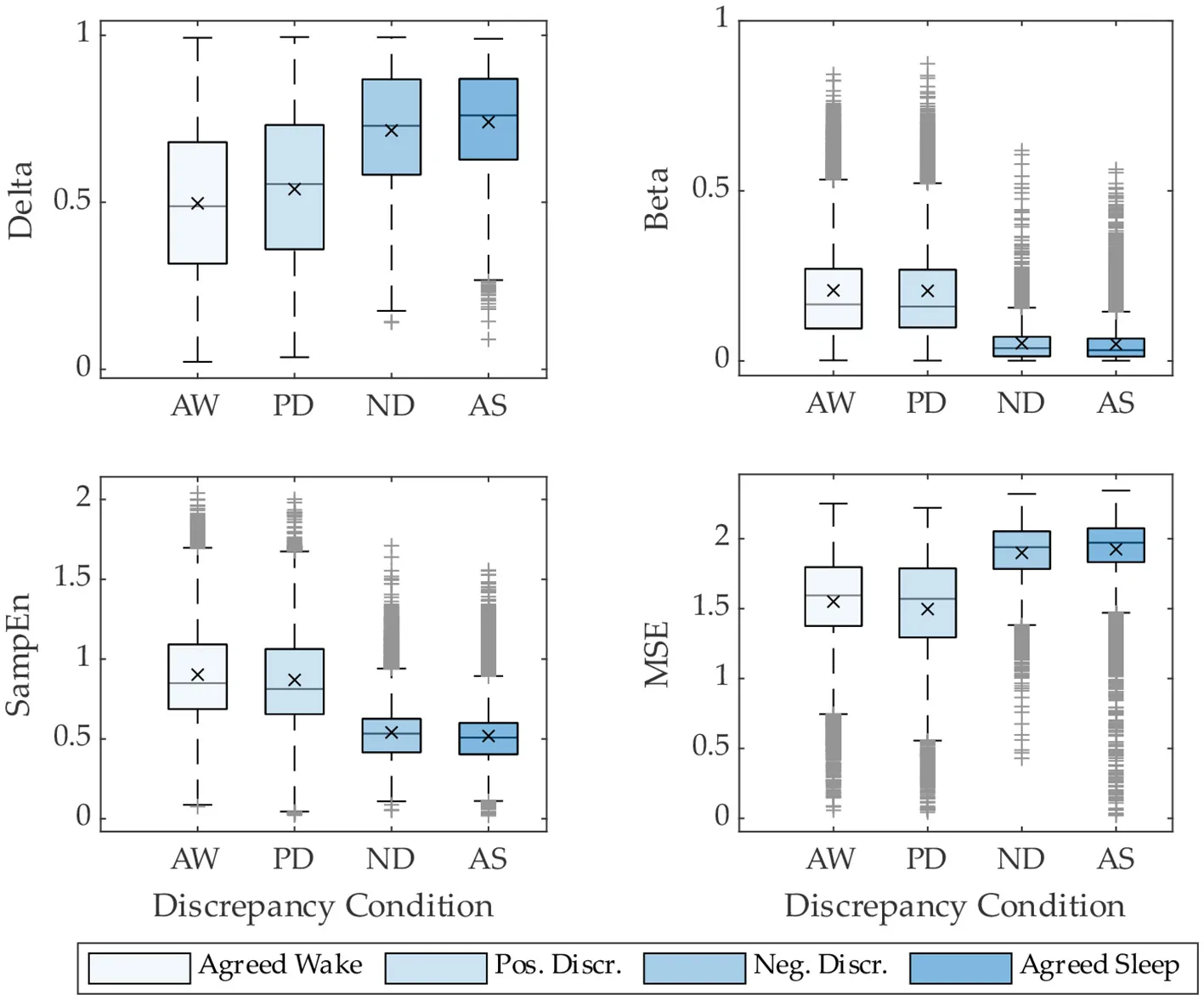
Relationship between EEG metrics (Delta, Beta, SampEn, and MSE) and discrepancy conditions (AW, agreed wake; PD, positive discrepancy; ND, negative discrepancy; AS, agreed sleep). The plots include data from the entire subject population. Lower and upper box edges represent the 25th and 75th percentile, respectively, and whisker lengths are 1.5 times the interquartile range. The ‘x' markers are the sample means.

A negative discrepancy, in which a participant reported wakefulness during physiologically measured sleep, represents a state associated with insomnia. It is therefore of interest to determine whether EEG metrics differ across discrepancy conditions, as shown in Figure 3.

Two key comparisons are AW vs. PD and ND vs. AS, which directly compare EEG measurements under equivalent PSG-defined sleep states. The results indicate that EEG metrics do not differ substantially between these conditions, suggesting that the metrics align more closely with physiological sleep states than with self-reports.

In contrast, comparisons of AW vs. ND and PD vs. AS, which reflect equivalent self-reported sleep states, show differences in EEG metrics. This suggests that, when relying on self-report alone, EEG metrics may help identify discrepancies and identify insomnia.

### 3.3 EEG metrics and insomnia status

**Figure 4:**
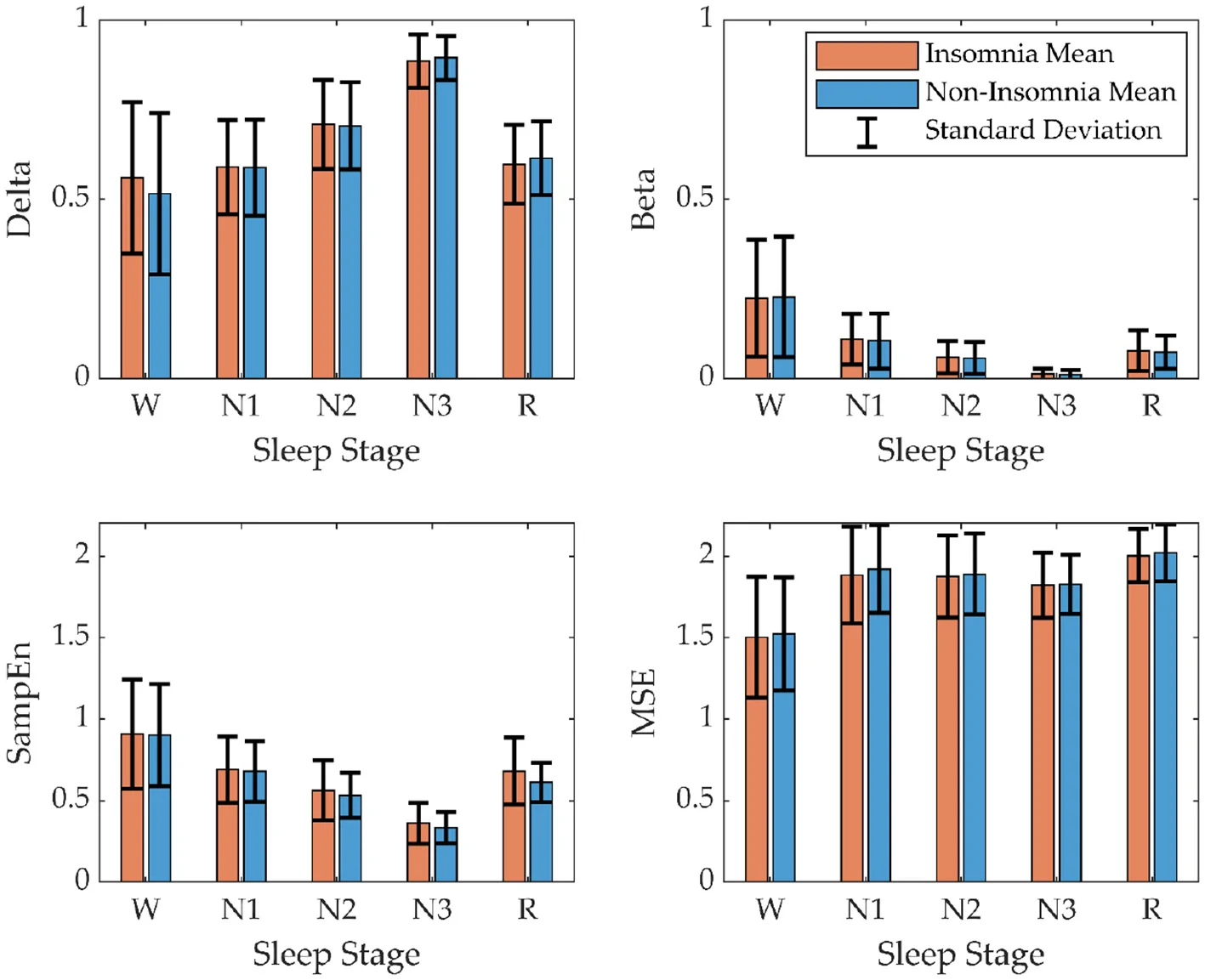
Comparison of EEG metrics (Delta, Beta, SampEn, and MSE) across sleep stages (W, N1, N2, N3, and R) for insomnia and non-insomnia groups. For the entire subject population, the bars represent the mean values of the metric at each sleep stage, and the error bars represent the standard deviation.

**Figure 5:**
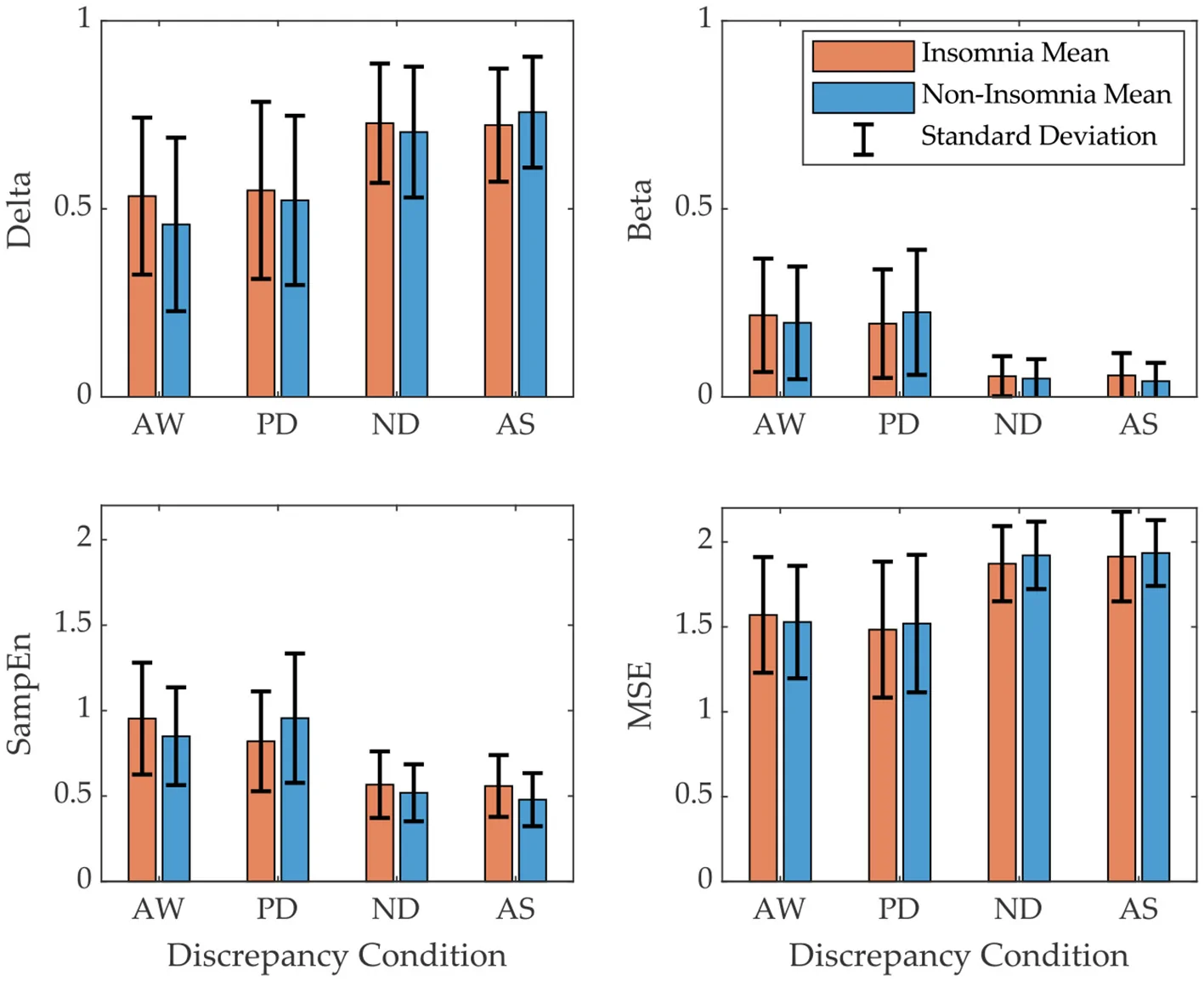
Comparison of EEG metrics (Delta, Beta, SampEn, and MSE) across discrepancy conditions (AW, agreed wake; PD, positive discrepancy; ND, negative discrepancy; AS, agreed sleep) for insomnia and non-insomnia groups. From the entire subject population, the bars represent the mean values of the metric at each sleep stage. The error bars represent standard deviation.

Figure 4 separates the data in Figure 2 by insomnia status to determine whether the mean EEG values differ between groups. Overall, mean band power and entropy values are modest. In the insomnia group, Delta power during stage W appears slightly higher, Beta power slightly lower, and SampEn slightly higher during REM sleep; however, the differences do not appear significant. Similarly, Figure 5 separates Figure 3 by insomnia status and displays similar patterns across discrepancy conditions.

**Figure 6:**
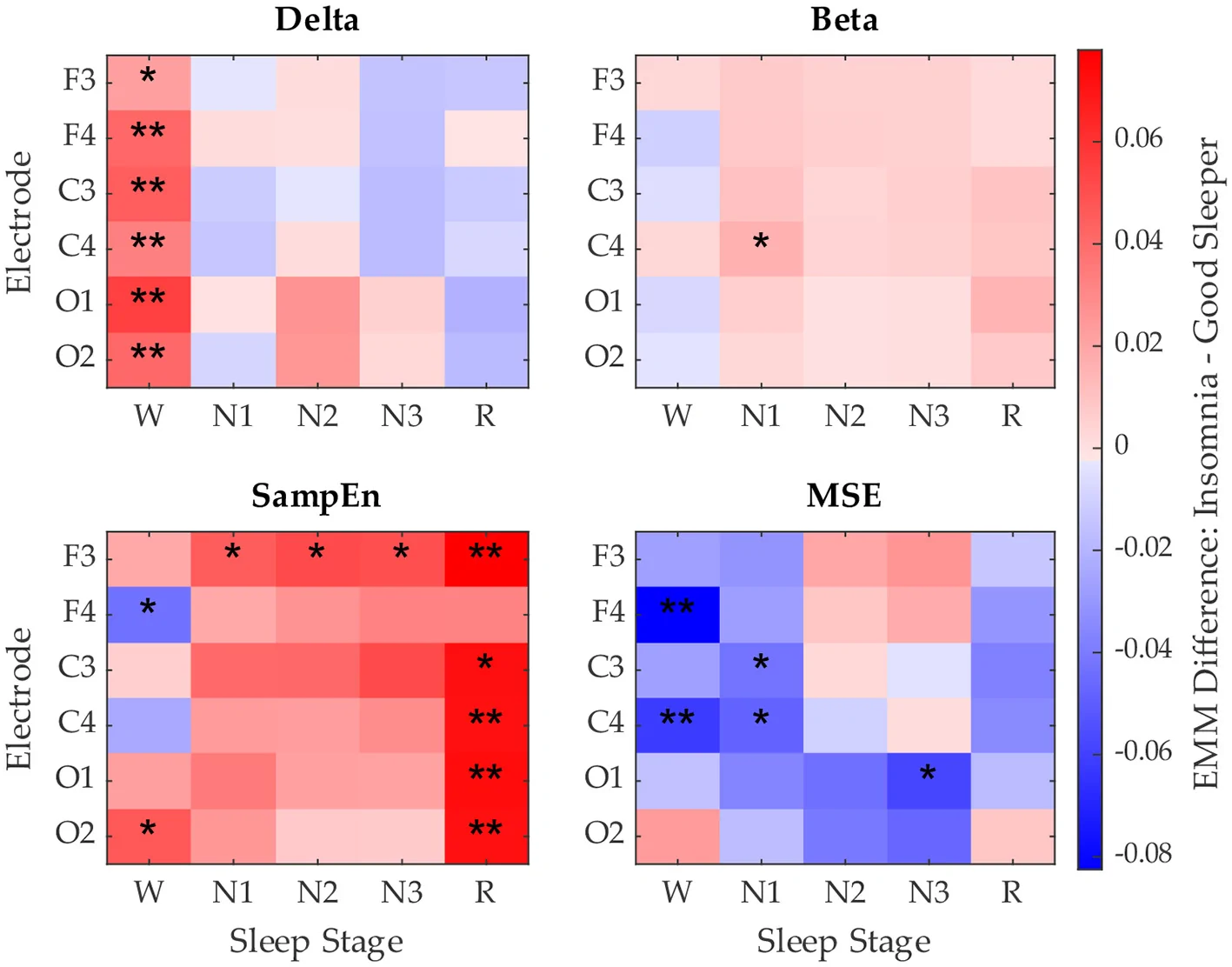
Heatmaps of estimated differences between insomnia and good sleeper data with significance markers (*p ≤ 0.05, **p ≤ 0.01) for sleep stages. Red cells indicate elevated estimates for individuals with insomnia compared to good sleepers.

**Figure 7:**
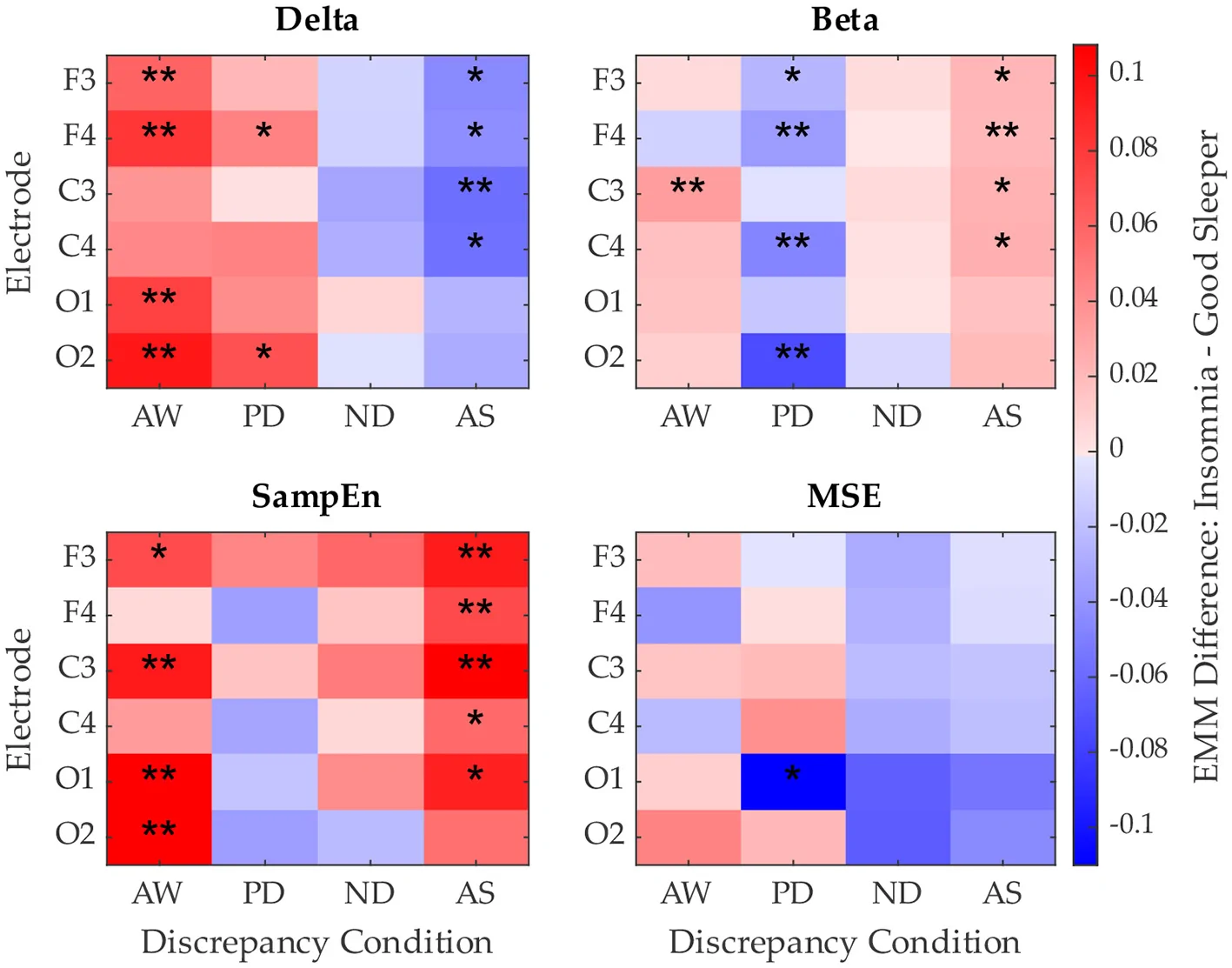
Heatmaps of estimated differences between insomnia and good sleeper data with significance markers (*p ≤ 0.05, **p ≤ 0.01) for discrepancy conditions (AW, agreed wake; PD, positive discrepancy; ND, negative discrepancy; AS, agreed sleep). Red cells indicate elevated estimates for individuals with insomnia compared to good sleepers.

If these EEG metrics are to correlate with sleep drive, arousal, or conscious awareness, it is not consistently distinguishable using these raw means data. Because comparisons may be influenced by non-normal distributions and variability within the data, linear mixed-effects modeling (LME) was used to identify statistically significant differences. These results are shown in Figures 6, 7.

The figures use a heat map style with a red-blue color scale to show differences within each metric, condition, and electrode location. Red cells indicate a higher estimate for those with insomnia compared to good sleepers, while blue cells represent a lower estimate. Cells where the model estimated a significant difference are marked with a single asterisk for *p* ≤ 0.05 and a double asterisk for *p* ≤ 0.01.

In Figure 6, individuals with insomnia show higher Delta power across all electrodes during wakefulness, while differences during sleep stages are smaller and less consistent. Beta power is generally lower during wakefulness and higher during sleep stages, although few differences reach statistical significance.

Continuing in Figure 6, SampEn shows mixed significance during stage W, being lower in the right frontal region (F4) and higher in the right occipital region (O2). In the left frontal region (F3), SampEn is significantly higher during all sleep stages, especially REM sleep. REM sleep also displays significantly higher SampEn in the central and occipital regions. Overall, SampEn trends higher in individuals with insomnia than in good sleepers, except for two electrodes during wakefulness.

MSE, representing complexity, is significantly lower among the insomnia group in some frontal and central regions during W and N1. During N2 and N3, MSE trends lower in the occipital regions but higher in the frontal and central regions, though not significantly (except for in channel O1 during N3). Finally, MSE drifts lower in five of six channels during REM sleep.

In Figure 7, the significant differences in Delta and SampEn during the AW condition align with the findings in Figure 6 during wakefulness. SampEn during AS also aligns with the previous findings. Despite the lack of significant differences in Delta and Beta power across sleep stages in Figures 6, 7 reveals some significant differences during the AS condition, which consists of non-REM sleep stages.

Considering the positive and negative discrepancy conditions, the most consistent significant difference observed relative to good sleepers is lower Beta power during the PD condition. Delta power is also generally higher during PD, though it reaches statistical significance at only two electrode locations (F4 and O2). Mixed results are observed across the remaining PD and ND conditions.

Since the magnitudes of these differences are relatively small, including those marked with an asterisk, practical significance was considered in addition to statistical significance using the 95% confidence intervals. For a given pairwise comparison, non-overlapping confidence intervals suggest a difference that is likely to be both statistically and practically meaningful. In contrast, statistically significant differences with overlapping confidence intervals may be less meaningful in a practical or clinical context. In the present study, all differences with significance level *p* ≤ 0.01 (double asterisk) were associated with non-overlapping confidence intervals and were therefore considered both statistically and practically meaningful.

## 4 Discussion

Overall, the findings indicate that EEG metrics correlate strongly with PSG-defined sleep stages and can distinguish sleep discrepancy when considered alongside self-reported sleep states. However, these metrics do not consistently differentiate individuals with insomnia from healthy sleepers across all contexts. However, notable differences were observed in Delta power and SampEn, which show results during sleep onset and REM sleep. The following discussion focuses on three aspects: (1) differences in EEG metrics across sleep stages between individuals with insomnia and good sleepers; (2) the relationship between EEG metrics and sleep discrepancies; and (3) the practical significance of the observed differences.

### 4.1 EEG metrics and sleep stages

EEG band powers and entropy metrics revealed patterns across sleep stages that are consistent with existing literature. From stages W to N3, increased Delta power and decreased Beta and SampEn reflect the transition from wakefulness to deep sleep, characterized by reduced high-frequency activity and increased neural synchronization (Gath, 1983; Bruce et al., 2009; Dressler et al., 2004). MSE showed a similar decreasing trend to Beta and SampEn during non-REM sleep but increased from W to N1. The decrease in MSE during sleep is consistent with previous studies of EEG complexity (Mateos et al., 2018; Mariani et al., 2016), including observations of higher complexity during N1 compared to wakefulness using MSE methods (Shi et al., 2017), which may correspond to the transition into low amplitude mixed frequency observed during N1 compared to the more consistent pattern of alpha waves during per-sleep wakefulness.

The correlation coefficients in Figure 1 further support these trends for Delta, Beta, and SampEn. For MSE, positive correlation coefficients initially suggest an increase across non-REM sleep stages; however, their relatively lower magnitudes (< 0.5) reflect the mixed pattern observed in Figure 2.

### 4.2 Regularity vs. complexity

The different trends observed between SampEn and MSE show that regularity and complexity are not necessarily synonymous (Costa et al., 2002; 2005; Goldberger et al., 2012). This can be understood by examining the underlying neural dynamics during different sleep stages. SampEn quantifies the irregularity of a signal in its original time scale. A lower SampEn during deep sleep indicates greater signal regularity due to synchronized neuronal activity (Costa et al., 2005; Li et al., 2015; Bruce et al., 2009). In contrast, MSE assesses signal complexity by computing SampEn across multiple time scales. For random signals, SampEn is low at large time scales due to reduction in variance as the smoothed signal approaches a constant value. Complex signals, such as physiological signals, maintain elevated SampEn due to long-range correlations and persistent variability despite the averaging of adjacent data points (Costa et al., 2002; 2005; Lau et al., 2022; Gao et al., 2015).

From stage W to N1, we observe a decrease in SampEn and an increase in MSE, demonstrating some independence between regularity and complexity. Alternatively, the low MSE of EEG data during wakefulness compared to sleep could reflect the presence of uncorrelated noise during wakefulness, allowing SampEn to decrease during sleep onset while MSE increases. From stages N1 through N3, both SampEn and MSE follow a downward trend until increasing again during REM sleep. The high complexity captured during light sleep and REM sleep in Figure 2 may be attributed to dynamic neural processing during sleep initiation and dreaming (Mizuseki and Miyawaki, 2019; Diekelmann and Born, 2010).

The divergence between decreasing regularity and increasing complexity highlights that these two metrics capture different aspects of the EEG signal. From a neurophysiological perspective, this pattern can be interpreted as the brain transitioning into a state where neuronal firing becomes more synchronized, leading to decreased irregularity, while interactions across neural assemblies remain intricate. During deep sleep, the brain engages in processes such as memory consolidation and synaptic homeostasis, which may involve coordinated activity across neural networks despite the overall increase in synchronization (Diekelmann and Born, 2010).

In the context of insomnia, SampEn and MSE could indicate alterations in either regularity or complexity of neural processes. For example, a smaller decrease in SampEn across sleep stages in individuals with insomnia compared to good sleepers could indicate impaired neuronal synchronization. Similarly, shifts in MSE might reflect disruptions in the complex interactions necessary for sleep initiation, sleep maintenance, or restorative sleep.

Regularity and complexity are not comprehensively discussed in this work [see (Mateos et al., 2018; Mariani et al., 2016; Shi et al., 2017; Lau et al., 2022)], but understanding the relationship between the two concepts enriches our interpretation of sleep physiology and its disturbances. It is important to use both single-scale and multiscale entropy measures for a more comprehensive picture of the brain's state during sleep.

### 4.3 EEG metrics and sleep discrepancy

Across sleep discrepancy conditions (Figure 3), the EEG metrics remained similar between conditions corresponding to PSG-defined wakefulness (AW and PD) and PSG-defined sleep (ND and AS). None of the EEG metrics significantly differentiated negative discrepancy from agreed sleep. These results suggest that the EEG metrics align closely with physiological sleep states rather than self-reported perceptions of sleep (Castelnovo et al., 2021).

In contrast, EEG metrics differed between conditions corresponding to self-reported wakefulness (AW and ND) and self-reported sleep (PD and AS), despite differences in PSG-defined sleep states. This indicates that EEG metrics may capture underlying physiological changes even when self-reported sleep state remains constant. This specifically applies to distinguishing perceived wakefulness from agreed wakefulness (ND from AW) and perceived sleep from agreed sleep (PD from AS).

Differences between PD and ND, as measured by the EEG metrics, can be compared across opposing self-reported sleep states to distinguish perceived sleep from perceived wakefulness. For example, an increase in Delta power, an established indicator of deepening sleep, occurring alongside a transition in self-report from sleep to wake indicates a discrepancy, as Delta power would otherwise be expected to decrease with increasing wakefulness.

### 4.4 EEG metrics and insomnia status

Comparing average EEG metrics between the insomnia and good sleeper groups revealed minor differences (Figures 4, 5). However, linear mixed-effects modeling identified some significant differences. Specific sleep stages and electrode locations of these findings are shown in Figure 6, and discrepancy conditions in Figure 7.

#### 4.4.1 PSG sleep stages

In Figure 6, the four EEG metrics, five sleep stages, and six electrode locations combine for a total of 120 pairwise comparisons between insomnia and good sleeper groups. Twenty-two instances (18%) were statistically significant with *p* ≤ 0.05 including 11 with *p* ≤ 0.01. The most significant differences during PSG sleep stages include Delta during wakefulness and SampEn during REM sleep. The higher Delta in insomnia during wakefulness may reflect increased sleep drive associated with local sleep deprivation in individuals with insomnia. Elevated SampEn during REM sleep may indicate heightened cortical arousal or conscious awareness in indviduals with insomnia.

Mixed results are observed in Beta and SampEn during wakefulness but are otherwise elevated for the insomnia group (red cells) across most sleep stages and electrodes. Although not significant in most cases, this pattern is generally consistent with previous studies suggesting elevated Beta activity in insomnia, particularly during sleep onset and light sleep (Perlis et al., 2001a,b). Intuitively, elevated Beta would be expected to correspond with reduced Delta activity when interpreted as wake drive and sleep drive, respectively. However, elevated Beta activity is accompanied by generally lower Delta during stages N1, N3, and R.

The mixed significance in SampEn between the right frontal and right occipital regions (F4 and O2) during wakefulness may suggest regional compensatory activity, where reduced irregularity in one region corresponds with increased irregularity in another. Similar patterns have been reported in other studies (Kay and Buysse, 2017; Levenson et al., 2015). The present study results suggest decreased SampEn in the right frontal region alongside increased SampEn in the right occipital region during wakefulness.

#### 4.4.2 Self-report sleep stages

In Figure 7, considering the positive and negative discrepancy conditions along with the four EEG metrics and six electrode locations, 48 pairwise comparisons were made. Five instances (10%) were statistically significant with *p* ≤ 0.05, including 3 with *p* ≤ 0.01. Many of the significant differences occurred during AW and AS, contributing to the PSG sleep stage observations. This pattern reflects greater variability and uncertainty in sleep stage classification during discrepancy conditions.

The most consistent and significant difference during discrepancy conditions was lower Beta power in the insomnia group during positive discrepancy. Interpreted in context, individuals with insomnia exhibited reduced high-frequency activity during epochs where they perceived sleep but were awake according to PSG. Differences observed during agreed wake and agreed sleep are consistent with previous findings (Figure 6), including elevated Delta during wakefulness and elevated SampEn during sleep. These results also provide additional evidence of elevated SampEn during agreed wake, which was less consistent in Figure 6.

#### 4.4.3 Limitations

Several limitations should be acknowledged. First, EEG signal processing and post-processing methods are not fully standardized beyond stage-scoring and filtering procedures established by the AASM (Berry et al., 2020). This is partly due to the wide range of PSG-related software, including many open-source programs. Automated exclusion of epochs with artifacts or noise is a critical step, and in this study, substantial in-house programming and data processing were required to balance efficiency with methodological control.

**Figure 8:**
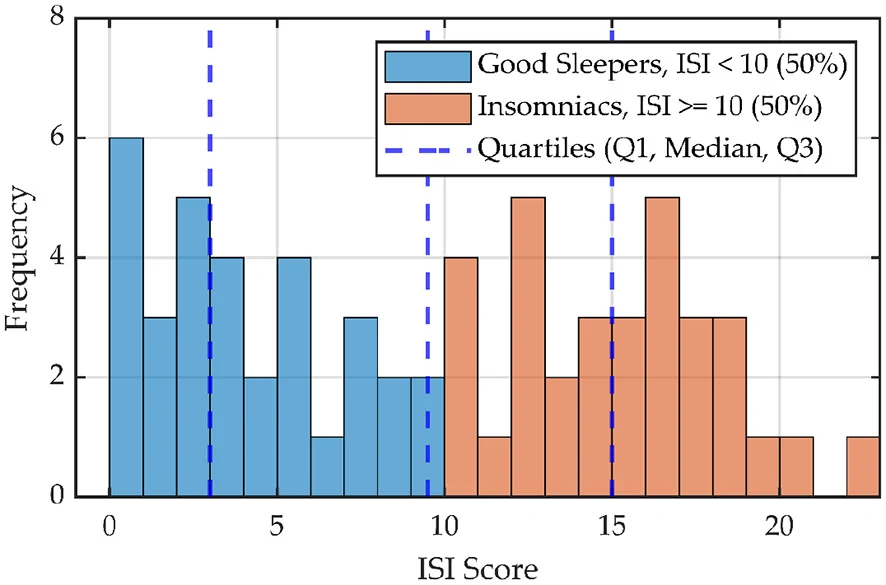
Histogram of insomnia severity index (ISI) scores.

Second, the distribution of insomnia severity index (ISI) scores among participants is an important consideration. Figure 8 shows a wide and relatively even distribution of insomnia severity, with the median near the classification threshold. Thirty-three of the 64 subjects fall within the interquartile range, while the remaining 31 subjects lie in the upper and lower quartiles, providing more definitive insomnia classifications. Although this distribution captures a broad range of insomnia experiences and provides useful context, a more polarized distribution may have allowed for clearer group comparisons.

Third, self-reported data and the derived measures such as ISI scores, in-bed times, sleep latency, and sleep discrepancy may lack accuracy. Several bedtimes were reported under different conditions, the values considered most reliable for this study were obtained from morning diary entries recorded upon waking. Additionally, the sleep discrepancy analysis was limited to the sleep onset period due to the absence of timestamped self-reported awakenings after sleep onset, potentially overlooking discrepancies occurring later in the night.

## 5 Conclusions

This study examined EEG band powers (Delta, Beta) and entropy metrics (SampEn, MSE) to identify neurophysiological differences between individuals with insomnia and good-sleeper controls across sleep stages and discrepancy conditions. These metrics correlate strongly with physiological sleep stages, although MSE exhibited a more variable pattern. Their ability to distinguish insomnia and good sleepers was limited; however, several characteristic differences were identified through sleep stage and discrepancy analyses.

During wakefulness, individuals with insomnia exhibited elevated Delta, decreased MSE, and mixed SampEn, suggesting increased sleep drive and potential compensatory activity. Notable trends were also observed during non-REM sleep, including generally elevated wake drive and conscious awareness (Beta and SampEn) in insomnia across electrodes, though relatively few differences reached statistical significance. Individuals with insomnia also showed increased SampEn during REM sleep, indicating heightened cortical arousal or conscious awareness.

EEG metrics did not differentiate discrepancy conditions when aligned with PSG-defined sleep stages, but were useful identifying discrepancies relative to self-report. Within positive and negative sleep discrepancy conditions, differences between insomnia and good sleeper groups were minimal, except for lower Beta and higher Delta during positive discrepancy. These findings suggest reduced arousal and increased sleep drive when PSG defined wakefulness is reported as sleep in individuals with insomnia. While previous studies that have linked negative sleep discrepancy with heightened EEG beta activity (Krystal et al., 2002; Perlis et al., 1997), our results did not consistently demonstrate this pattern. This highlights the challenges in consistently detecting heightened arousal in individuals with insomnia using standard EEG metrics. Insomnia may also be reflected by diminished arousal during wakefulness. These findings emphasize the importance of integrating both physiological and self-report measures to better characterize insomnia, particularly during discrepancy conditions.

This study contributes to the existing body of work by examining entropy measures (SampEn and MSE) alongside spectral power across sleep stages and discrepancy conditions. These findings highlight that standard EEG metrics alone do not provide a consistent biomarker of insomnia, but they capture meaningful aspects of sleep drive, arousal, and conscious awareness under specific conditions. Future work that seeks to integrate physiological and self-reported measures may improve the characterization of insomnia-related neurophysiological changes.

## Data Availability

The original contributions presented in the study are included in the article/supplementary material, further inquiries can be directed to the corresponding author/s.

## References

[B1] Berry R. B. Quan S. Abreu A. (2020). The AAS M Manual for the Scoring of Sleep and Associated Events: Rules Terminology and Technical Specifications Version 2.6. Darien, IL: American Academy of Sleep Medicine.

[B2] Bruce E. N. Bruce M. C. Vennelaganti S. (2009). Sample entropy tracks changes in electroencephalogram power spectrum with sleep state and aging. J. Clin. Neurophysiol. 26, 257–266. doi: 10.1097/WNP.0b013e3181b2f1e3 19590434 PMC2736605

[B3] Buzsákí G. Draguhn A. (2004). Review neuronal oscillations in cortical networks. Available online at: www.sciencemag.org doi: 10.1126/science.1099745 (Accessed December 2025). 15218136

[B4] Castelnovo A. Ferri R. Galbiati A. Rossi A. Zucconi M. Castonovo V. . (2021). Extreme sleep state misperception: from psychopathology to objective-subjective sleep measures. Int. J. Psychophysiol. 167, 77–85. doi: 10.1016/j.ijpsycho.2021.06.011 34216692

[B5] Costa M. Goldberger A. L. Peng C. K. (2002). Multiscale entropy analysis of complex physiologic time series. Phys. Rev. Lett. 89. doi: 10.1103/PhysRevLett.89.068102 12190613

[B6] Costa M. Goldberger A. L. Peng C. K. (2005). Multiscale entropy analysis of biological signals. Phys. Rev. E. Stat. Nonlin. Soft Matter. Phys. 71. doi: 10.1103/PhysRevE.71.021906 15783351

[B7] Diekelmann S. Born J. (2010). The memory function of sleep. Nature 11, 114–126. doi: 10.1038/nrn2762 20046194

[B8] Dressler O. Schneider G. Stockmanns G. Kochs E. F. (2004). Awareness and the EEG power spectrum: analysis of frequencies. Br. J. Anaesth. 93, 806–809. doi: 10.1093/bja/aeh270 15377585

[B9] Gao J. Hu J. Liu F. Cao Y. (2015). Multiscale entropy analysis of biological signals: a fundamental bi-scaling law. Front. Comput. Neurosci. 9. doi: 10.3389/fncom.2015.00064 26082711 PMC4451367

[B10] Gath I. (1983). Bar-on classical sleep stages and the spectral content of the EEG signal. Int. J. Neurosci. 22, 147–155. doi: 10.3109/00207459308987393 6668131

[B11] Goldberger A. L. Moody G. B. Costa M. D. (2012). Variability vs. complexity. Available online at: https://archive.physionet.org/tutorials/cv/ (Accessed December, 2025).

[B12] Himes B. J. Blotter J. D. Kay D. B. Feland J. B. Steffensen S. C. (2020). Development and analysis of a vibration based sleep improvement device. Provo, UT: Brigham Young University.

[B13] Himes B. J. Blotter J. D. Kay D. B. Steffensen S. C. (2021). The effect of beat frequency vibration on sleep latency and neural complexity: a pilot study. IEEE Trans. Neural Syst. Rehabil. Eng. 29, 872–883. doi: 10.1109/TNSRE.2021.3076983 33950842

[B14] Kay D. B. Buysse D. J. (2017). Hyperarousal and Beyond: New Insights to the Pathophysiology of Insomnia Disorder Through Functional Neuroimaging Studies. MDPI AG. doi: 10.3390/brainsci7030023 28241468 PMC5366822

[B15] Krystal A. D. Edinger J. D. Wohlgemuth W. K. Marsh G. R. (2002). NREM Sleep EEG frequency spectral correlates of sleep complaints in primary insomnia subtypes. Available online at: https://academic.oup.com/sleep/article/25/6/626/2750129 (Accessed December, 2025). 12224842

[B16] Lau Z. J. Pham T. Chen S. H. A. Makowski D. (2022). Brain Entropy, Fractal Dimensions and Predictability: A Review of Complexity Measures for EEG in Healthy and Neuropsychiatric Populations. John Wiley and Sons Inc. doi: 10.1111/ejn.15800 35985344 PMC9826422

[B17] Levenson J. C. Kay D. B. Buysse D. J. (2015).The pathophysiology of insomnia. Chest 147, 1179–1192. doi: 10.1378/chest.14-1617 25846534 PMC4388122

[B18] Li H. Peng C. Ye D. (2015). A study of sleep staging based on a sample entropy analysis of electroencephalogram. Biomed. Mater. Eng. 26. S1149–S1156. doi: 10.3233/BME-151411 26405872

[B19] Malik J. (2024). Multiscale sample entropy. MATLAB Central File Exchange: 1.3.0.0. Available online at: https://www.mathworks.com/matlabcentral/fileexchange/62706-multiscale-sample-entropy (Accessed May 21, 2022).

[B20] Mariani S. Borges A. F. Henriques T. Thomas R. J. Leistedt S. J. Linkowski P. . (2016). Analysis of the sleep EEG in the complexity domain,” in *Proceedings of the Annual International Conference of the IEEE Engineering in Medicine and Biology Society EMBS Institute of Electrical and Electronics Engineers, Inc*., 6429–6432. doi: 10.1109/EMBC.2016.7592200 28269718 PMC5501079

[B21] Mateos D. M. Guevara Erra R. Wennberg R. Perez Velazquez J. L. (2018). Measures of entropy and complexity in altered states of consciousness. Cogn. Neurodyn. 12, 73–84. doi: 10.1007/s11571-017-9459-8 29435088 PMC5801282

[B22] Mizuseki K. Miyawaki H. (2019). “Hippocampal information processing and homeostatic regulation during REM and non-REM sleep,” in Handbook of Behavioral Neuroscience, Vol. 30 ed. Diba K. (Elsevier B.V.), 49–62. doi: 10.1016/B978-0-12-813743-7.00004-9

[B23] Niedermeyer E. Fernando L. D. S. (2004). Electroencephalography: Basic Principles Clinical Applications and Related Fields, 5th Edn. Philadelphia, PA: WoltersKluwer.

[B24] Perlis M. L. Giles D. E. Mendelson W. B. Bootzin R. R. Wyatt J. K. (1997). Psychophysiological Insomnia: The Behavioural Model and a Neurocognitive Perspective. Blackwell Publishing Ltd. doi: 10.1046/j.1365-2869.1997.00045.x 9358396

[B25] Perlis M. L. Merica H. Smith M. T. Giles D. E. (2001a). Beta EEG Activity and Insomnia. W.B. Saunders Ltd. doi: 10.1053/smrv.2001.0151 12531000

[B26] Perlis M. L. Smith M. T. Andrews P. J. Orff H. Giles D. E. (2001b). Beta/Gamma EEG activity in patients with primary and secondary, insomnia and good sleeper controls Beta/Gamma EEG activity in patients with insomnia. doi: 10.1093/sleep/24.1.110 11204046

[B27] Shi W. Shang P. Ma Y. Sun S. Yeh C. H. (2017). A comparison study on stages of sleep: quantifying multiscale complexity using higher moments on coarse-graining. Commun. Nonlinear Sci. Numer. Simul. 44, 292–303. doi: 10.1016/j.cnsns.2016.08.019

[B28] Zhao W. Van Someren E. J. W. Li C. Gui W. Tian Y. Liu Y . (2021). EEG spectral analysis in insomnia disorder: a systematic review and meta-analyisis. Sleep Medicine Rev. 59:101457. doi: 10.1016/j.smrv.2021.101457 33607464

